# Colors in the dark

**DOI:** 10.1093/plphys/kiac140

**Published:** 2022-03-24

**Authors:** Miriam Oses-Ruiz

**Affiliations:** Institute for Multidisciplinary Applied Biology (IMAB), Public University of Navarre (UPNA), Campus Arrosadia, 31006 Pamplona, Spain

Plants have the ability to regulate their growth and development according to available light (Li et al., 2012). Light perception occurs through photoreceptors, such as phytochromes, cryptochromes, and phototropins, that translate the signal inside cells where arrays of transcription factors repress or activate genes required for cellular processes (Kong and Okajima, 2016; Mawphlang and Kharshiing, 2017). One of these processes is the regulation of pigment synthesis, including chlorophylls and carotenoids.

In most plants, carotenoid biosynthesis depends tightly on light responses where light triggers carotenoid production and darkness represses it. One of the best-known examples occurs in the model plant Arabidopsis (*Arabidopsis thaliana*). In dark conditions, basic helix–loop–helix (bHLH) proteins called PHYTOCHROME INTERACTING FACTORS (PIFs) accumulate to bind and repress transcription from light response elements. In this way, photomorphogenesis is repressed, including the expression of PHYTOENE SYNTHASE (*PSY*) gene, which encodes the first enzyme in the carotenoid biosynthesis pathway (Von Lintig et al., 1997). In light conditions, PIFs are phosphorylated and therefore triggered for degradation, releasing *PSY* expression from inhibition and causing carotenoid biosynthesis to occur.

In carrot (*Daucus carota*), the molecular scenario must be very different as carrots accumulate carotenoids in the roots during dark conditions. Carrots are one of the vegetables that accumulate the most carotenoids, alongside mint and parsley (Qudah, 2008). How carotenoid biosynthesis occurs in the dark in carrot roots and what other components are involved are not well understood. Carotenoids have antioxidant and pro-vitamin A activity and provide nutritional value for human diets (Alós et al., 2016). Carotenoids are stored in chromoplasts, which differentiate from other types of plastids such as chloroplasts (Egea et al., 2010). Light/dark balance affects chromoplast differentiation and carotenoid accumulation (Egea et al., 2010), but both processes remain still somewhat unknown. Understanding how carotenoid biosynthesis occurs at the molecular level is vital to design strategies to increase carotenoid content and improve nutritional properties of carrots and other crops (Alós et al., 2016). One of the best-known examples of genetic modification leading to an increase in carotenoid content is “golden rice,” where carotenoid content was increased by manipulating and using the transgenes *PSY* and *CTRI* (CAROTENE DESATURASE) (Xudong et al., 2000). Genetically manipulating carotenoid biosynthesis pathways for accumulation of this pigment is pivotal to rapidly provide food that can help with human health issues, such as blindness due to VAD (Gayen et al., 2016).

**Figure 1:**
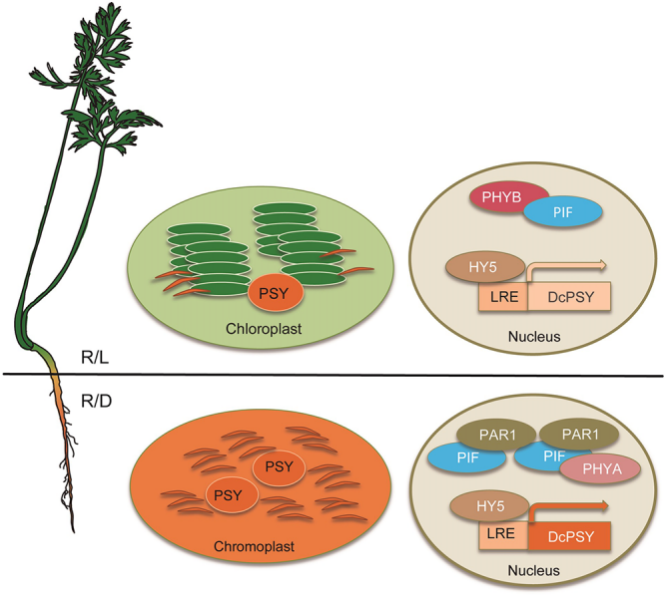
Model proposed by Arias et al. (2022) showing the role of the bHLH protein PAR1 in regulating carotenoid biosynthesis during darkness in carrot roots. During light conditions, PAR1 is not produced and therefore *PSY* (PHYTOENE SYNTHASE) gene expression and carotenoid biosynthesis is low. Under darkness, *PAR1* expression is triggered, which causes an increase in transcripts and protein levels of *PSY* and therefore carotenoids accumulate (image from Arias et al., 2022). R/L, roots grown in light; R/D, roots grown in darkness; PHYA, PHYTOCHROME A; PHYB, PHYTOCHROME B; and HY5: LONG HYPOCOTYL.

In this issue of *Plant Physiology*, Arias et al. (2022) investigated how carotenoid biosynthesis occurs and how is it regulated in dark conditions by elucidating the role of the bHLH protein PHYTOCHROME RAPIDLY REGULATED 1 (PAR1). PAR1 was previously noted to be of interest from an RNA-seq analysis where gene expression was compared between carrot roots when grown in white light versus darkness, revealing a set of dark-expressed photomorphogenesis-related genes, including *PHYA* (PHYTOCHROME A), *PIF4*, and *PAR1* (Arias et al., 2020)*.* In Arabidopsis, *PAR1* is a cofactor that likely associates with PIFs to promote carotenoid accumulation (Bou-Torrent et al., 2015). The exact role of PAR1 in carrot plants remains to be elucidated (Figure 1).

The authors identified a *PAR1* ortholog in Arabidopsis and expressed this gene in carrots. *AtPAR1* overexpression triggered increased carotenoid levels and *PSY1* expression. This suggested *PAR1* could be the regulator of carotenoid biosynthesis. When the authors carried out the converse experiment and expressed *D.* *carota PAR1* in Arabidopsis, carotenoid levels again increased due to increased *PSY* gene expression and protein abundance. This suggested a relationship between *PAR1* and *PSY* regulates carotenoid biosynthesis.

To investigate this scenario further, the authors carried out several experiments. First, the authors designed carrot plants with reduced *PAR1* expression. Carotenoid content in 4- and 8-month-old transgenic plants decreased, confirming that *PAR1* is required for carotenoid accumulation. Next, the authors demonstrated that carotenoid accumulation in *PAR1*-silenced plants decreased because genes required for carotenoid biosynthesis, such as *PSY1*, *PSY2*, *LCYE* (LYCOPENE Ε-CYCLASE), *LCYB2* (LYCOPENE β-CYCLASE), *CHXB1*, and *CHXB2* (β-RING CAROTEN HYDROXYLASE), were downregulated. The authors hypothesized that this might occur via an interaction of PAR1 with other transcription factors, such as PIFs. Finally, the authors found increased chlorophyll and lutein content in *PAR1*-silenced plants. This suggested that the absence of *PAR1* might contribute to chloroplast formation. Whether *PAR1* could be involved not only in carotenoid biosynthesis but also in chromoplast formation remains to be investigated further.

Altogether this study showed that increased gene expression of the bHLH protein PAR1 in carrot plants in dark conditions induces the expression of carotenoid biosynthesis genes, including the phytoene synthase enzyme that triggers carotenoid accumulation in the chromoplast. The interconnection between lutein and chlorophyll biosynthesis and carotenoid biosynthesis and accessory partners of *PAR1* that play a role in fine-tuning carotenoid biosynthesis in carrots requires further investigation.

*Conflict of interest statement.* None declared.
